# A Review on Assisted Living Using Wearable Devices

**DOI:** 10.3390/s24237439

**Published:** 2024-11-21

**Authors:** Grazia Iadarola, Alessandro Mengarelli, Paolo Crippa, Sandro Fioretti, Susanna Spinsante

**Affiliations:** Dipartimento di Ingegneria dell’Informazione, Università Politecnica delle Marche, 60131 Ancona, Italy; g.iadarola@staff.univpm.it (G.I.); a.mengarelli@staff.univpm.it (A.M.); p.crippa@staff.univpm.it (P.C.); s.fioretti@staff.univpm.it (S.F.)

**Keywords:** wearable devices, assisted living, human activity recognition, electromyography, skin conductance, biomedical measurement systems

## Abstract

Forecasts about the aging trend of the world population agree on identifying increased life expectancy as a serious risk factor for the financial sustainability of social healthcare systems if not properly supported by innovative care management policies. Such policies should include the integration within traditional healthcare services of assistive technologies as tools for prolonging healthy and independent living at home, but also for introducing innovations in clinical practice such as long-term and remote health monitoring. For their part, solutions for active and assisted living have now reached a high degree of technological maturity, thanks to the considerable amount of research work carried out in recent years to develop highly reliable and energy-efficient wearable sensors capable of enabling the development of systems to monitor activity and physiological parameters over time, and in a minimally invasive manner. This work reviews the role of wearable sensors in the design and development of assisted living solutions, focusing on human activity recognition by joint use of onboard electromyography sensors and inertial measurement units and on the acquisition of parameters related to overall physical and psychological conditions, such as heart activity and skin conductance.

## 1. Introduction

According to the “2024 Revision of World Population Prospects” published by the United Nations [1], the total global population aged 65 and older is projected to reach 2.2 billion, surpassing the number of children (under age 18) by the late 2070s, and a similar increasing trend will be observed for the population aged 80 and over, which is projected to reach 265 million as early as the mid-2030s. This aging of people has an ever-increasing impact on families, communities, and societies in most countries worldwide. While, on the one hand, a longer life expectancy is the result of social progress and impressive improvements in medicine and healthcare, on the other hand, the rate of older adults living longer but losing their autonomy because of poor health conditions has a relevant impact on the financial sustainability of modern public healthcare and welfare systems [2,3]. Therefore, ever-increasing efforts and new approaches are needed to assist the independent daily lives of the elderly, resulting in a growing interest in research and development of methods, technologies, and solutions for active and assisted living (AAL) [4].

Among the chronic conditions that deeply affect the quality of life of older adults, it is worth mentioning age-related neural diseases, such as Parkinson’s and Alzheimer’s diseases. They are often characterized by a progressive, and sometimes quick, decline in functional and cognitive abilities. Therefore, ensuring independent living for people suffering from these diseases is very difficult, especially due to the progressive behavioral and psychiatric symptoms that are often present in affected subjects. As a result, caring for these individuals is particularly stressful, and the quality of life experienced by family caregivers is strongly compromised. Since a lower quality of life often translates into increased absences from work and reduced work productivity, it is very important to identify devices and systems that can help and support caregivers in the daily activities of caring. This way, the quality of life of people in need of assistance and those who care for them will improve, and the social costs linked to diseases will be reduced [5,6]. Needs expressed by caregivers of people affected by dementia or related diseases pertain to the physical, psychological, and social demands of providing assistance [7]. Among them, it is of paramount importance to provide caregivers with assistive devices and systems that can help detect potentially harmful, unsafe, or dangerous activities in order to generate alerts and prevent accidents. Human activity recognition (HAR) is an engineering research area devoted to the implementation of devices, systems, and methods for the automatic recognition and classification of human activities based on sensor data. It has been demonstrated that HAR applied to simple household activities (e.g., sleeping or walking, going up or down the stairs, and opening or closing doors) as well as to complex activities (e.g., riding a bicycle or driving a car) can strongly support the surveillance, care, and treatment of the elderly or people suffering from age-related diseases [8]. In addition to the above considerations, HAR also plays a fundamental role in the design and assessment of assistive robotic devices, dealing with locomotion and other body movements [9], which are important components of modern AAL systems.

In a similar manner, AAL strongly relies on the capability of monitoring people’s conditions, i.e., physical, mental, and emotional, by the continuous, long-term, and minimally invasive acquisition of health-related signals, from which quantitative parameters can be extracted. The latter may be of huge importance, for example, in checking the progression of diseases, evaluating the steadiness of physical and cognitive functionalities, and detecting acute episodes that could lead to a decrease in autonomy and quality of independent living. In this context, modern wearable devices can help break the paradigm of traditional medicine based on single visits, scattered over time, to clinics and health facilities for the execution of specific tests by healthcare operators, using clinical instruments in controlled environments instead to promote approaches based on long-term multidimensional observations in living environments and in everyday life conditions. The data collected from wearable devices include a variety of body signals that can aid in the implementation of personalized interventions, supporting preventive measures and lifestyle modifications against the development of chronic conditions limiting elderly well-being [10]. However, some aspects that can still limit the contribution that wearable devices are able to provide to the development of AAL systems must be considered, in particular with regard to the quality of the signals acquired in the presence of disturbances determined by the conditions of use, the environment, and the lack of industry standards [11].

In this work, the attention is focused on the possibilities opened up by wearable devices for the implementation of effective and user-acceptable AAL solutions by combining different embedded sensors made available by recent developments in electronic design. Starting from Section 2, we provide an overview of the electronic components and sensors found in wearable devices, such as accelerometers and sensors for the acquisition of surface electromyographic (sEMG) and other body signals, also looking at commercially available devices. In Section 3, the related processing approaches enabling the monitoring of both activity recognition and physiological parameters by the integrated use of sensors, with different scopes and applications, are presented. Section 4 provides an overall discussion of the role of wearable devices in AAL, and finally, Section 5 draws the main conclusions of the work.

## 2. Wearable Devices Enabling AAL Solutions

AAL solutions are based on information and communication technology (ICT) systems to support and enhance the quality of life and well-being of older or impaired people in both indoor and outdoor environments using a distributed network of sensors and actuators to maintain the people’s independence and autonomy, while ensuring safety and providing assistance. HAR is considered a pivotal technology within the field of AAL in that it is responsible for the automatic detection and classification of the activities performed by individuals using sensor-based systems. Researchers and engineers have exploited cutting-edge advances in micro- and nano-electronics, flexible and integrated circuits, signal processing, and communication network protocols to create small, low-power, and low-cost sensors that can be easily worn or placed directly on the body skin to acquire large amounts of data, such as inertial body information, heart rate (HR), muscle fatigue, skin conductance (SC), and so on [12,13,14]. These tiny sensors and devices may even power themselves from human motion while sensing [15].

### 2.1. Acceleration Sensors

The simplest way to recognize human activities is to acquire body speed and orientation based on accelerometers placed on the body. Often, in embedded systems, accelerometers are augmented with gyroscopes that measure the angular velocity along three axes, which can be used to increase the accuracy of electronic compasses and improve the estimate of the orientation, otherwise performed only with the accelerometer.

Usually, for a given set of activities of interest in AAL applications, a single accelerometer sensor, positioned on the wrist, ankle, or alternatively embedded in a smartwatch, may be sufficient to monitor elderly living at home or in residential centers [16,17,18,19,20] in a very simple manner. In the last decade, the huge development of the global smartwatch market has made wearable and autonomous accelerometer sensors available everywhere and to everyone. Many HAR systems have been developed by exploiting data acquired by these personal devices, and the placement of multiple accelerometer sensors on the body has been considered as well [21].

More details about the experimental setup used in the above-mentioned studies, together with some details about the sensor characteristics, like full scale and sampling frequency, are given in Table 1. In the case of elderly people, these systems could be used to detect alarming conditions generated by the unusual behavior of the person (inability to get out of bed, absence of activity for a defined period of time) or changes in routine activities related to psychomotor or neurodegenerative diseases. In applications dealing with rehabilitation, these systems could be used to monitor training routines, e.g., to count repetitions of exercises and determine the fatigue or energy expenditure associated with individual movements.

### 2.2. EMG Sensors

The surface electromyography (sEMG) signal, generated by the electrical potential of muscle contractions, is one of the easiest biological signals to acquire using non-invasive sensor systems; therefore, it has proven to be very useful in monitoring muscle strength, health status, fitness levels, and physical performance [22,23,24,25,26].

The sensors used to acquire sEMG signals can easily be derived from those used for acquiring ECG signals [27,28,29], even if they are quite different from those used to detect cardiac activity [30] due mainly to the larger bandwidth of the EMG signals that have a frequency content that extends up to 500 Hz [31] and is more likely to be acquired during movements, being therefore affected by artifacts induced by body and cable movements at frequencies usually below 5 Hz. This must be taken into account when designing sensors capable of acquiring both EMG and ECG signals [32]. In particular, low-frequency motion artifacts (MAs) must be rejected by the high-input-impedance low-noise amplifier, which should be carefully designed to capture the relatively low amplitude of the sEMG signal without incurring saturation [28,33,34]. Furthermore, data derived from integrated triaxial accelerometers can be used to reduce the MAs [35] and improve the performance of the sEMG acquisition node when used for activity detection and classification.

Innovative and diverse health monitoring systems and interfaces [36] have been made possible thanks to advances in several design aspects, such as micro- and flexible electronic components [37], new nanotechnologies, tiny wireless communication interfaces, as well as powerful but lightweight machine learning algorithms [38,39,40]. Body data acquisition has become easier, more affordable, and reliable thanks to sensors that, being small, ultra-low-power, and wireless-enabled, can be embedded into small devices (smartwatches, smartphones, wristbands, rings, and glasses), clothing, or directly placed on the skin [27,41,42]. For these reasons, healthcare and AAL domains have started to increasingly use and rely on wearable sensors [43]. As an example, in [44], a wearable, lightweight, and low-cost sEMG sensor is implemented in combination with a machine learning classifier to identify neck postural risks caused by prolonged work or human behavior involving upper extremities. Furthermore, in [45], a wearable system using an e-textile substrate combines signals from sEMG sensors with inertial data from IMUs acquired from the cervical region to monitor in real-time the occurrence of forward head posture that is a habitual bad posture of the neck, consisting of the forward translation of the cervical vertebrae and hyperextension of the upper ones.

Table 2 provides a summary of studies involving the development and use of miniaturized multimodal sensors, often implemented as systems-on-chip (SoCs), for different acquisition and monitoring purposes.

Typically, wearable sEMG sensing nodes for AAL applications must efficiently transmit the acquired data to base stations wirelessly using low-power and possibly low-cost solutions. Therefore, several wireless EMG signal acquisition systems use 2.4 GHz radio links based on the IEEE 802.15.4 standard protocol [56] to transmit their data [57,58], exploiting the well-known ZigBee technology. In [28], a low-cost wearable system based on several ultralight wireless sensor nodes has been proposed to acquire and process EMG, ECG, and acceleration signals and transmit data toward base stations through a custom communication protocol designed on the IEEE 802.15.4 physical layer to exploit current technology and improve the throughput and synchronization allowed by the standard. In [47,59], a three-channel wireless EMG/ECG/IMU sensor using a Bluetooth Low Energy (BLE) radio for real-time data transmission has been proposed in a basic hardware configuration to minimize power consumption. Real-time operation is ensured by an adaptation layer on top of BLE that enables tight time synchronization and reliability of data transmission, while energy efficiency is ensured by motion-triggered wake-up capability, thanks to the onboard inertial measurement unit (IMU) that switches on the system only when worn.

### 2.3. Skin Conductance Sensors

The human skin exhibits time-varying electrical characteristics, as captured by a continuous-time signal known as skin conductance (SC), or, alternatively, the galvanic skin response (GSR), i.e., the reciprocal of skin conductance, namely skin resistance [60], or electrodermal activity (EDA). Those changes in the skin’s electrical properties are due to body sweating [61], which indicates psychological or physiological arousal reflecting people’s emotions. Physiology underneath classical approaches to SC analysis relates SC variations to the status of sweat glands spread over the skin and regulated by the autonomic nervous system (ANS): high arousal of the ANS sympathetic branch increases the activity of the sweat glands, thus enhancing SC. Conversely, elevated sweat gland activity indicates both physiological and psychological arousal, which may be captured by changes in the SC. On the other hand, the activity of sweat glands is autonomously influenced by the sympathetic nervous system (SNS) branch driving subconscious human behavior so that the SC signal may be exploited by devices aimed at supporting meditation, monitoring stress, and the overall physical and psychological condition of a subject [62].

Given these characteristics of human physiology, it can be observed that slow changes of the SC, known as the tonic level or skin conductance level (SCL), mostly depend on skin dryness, hydration, and automatic body temperature regulation, while rapid variations of the SC, i.e., the so-called phasic level or skin conductance response (SCR), represent dynamic changes associated with the reaction of a subject to different stimuli, either physical, emotional, or cognitive [61], thus reflecting their psychological [63] and emotional status. SC has been discovered to have a positive correlation with various affective and cognitive encounters, such as stress, anxiety, fear, physical exertion, attention, and high cognitive load. As a result, SC serves as a readily available and sensitive indicator of sympathetic drive, making it an ideal measure of psychophysiological arousal in a conscious and healthy individual [64].

While it is possible to obtain direct recordings of sympathetic nerve bundles by inserting a thin microelectrode under the skin, more comfortable and non-invasive methods are preferred for longitudinal recordings. One such method involves passing a small external current through two electrodes (dry or wet) placed on the skin’s surface and calculating electrical conductance, which is the reciprocal of resistance. Electrodes are typically located on the second and third fingertips of the same hand (usually the dominant one). The variations of the voltage generated by a small applied current circulating through the skin between the electrodes provide a measure of the SC. Such a measuring approach is implemented in laboratory equipment, where a proper DAQ ensures quite a high sampling frequency (typically, 1000 Hz [65]). With the recent advent of comfortable-to-wear commercial healthcare devices, usually designed as bracelets, watches, or rings, the SC measure has also become available in uncontrolled settings to support research in the neuroscience domain. In this method, referred to as exosomatic, an external energy source, such as a constant voltage or current, is employed. The signals detected primarily arise from alterations in resistance and allow for an estimation of increases in sympathetic nerve activity, even in the absence of observable sweating. It is worth noting that both the skin and sweat glands possess resistive and capacitive properties that can be effectively modeled [66]. It is important to note that there are various other techniques available for measuring electrodermal activity. However, the most extensively studied and wearable approach involves the method discussed above. Typically, researchers measure and report the SCL, which represents a slow-moving average of the electrodermal activity signal over time. Additionally, they also analyze SCRs, which capture more rapid fluctuations and phasic changes in the activity [64,67].

Serving as a crucial link between the brain and behavior, peripheral physiological signals offer valuable insights into the workings of the body and mind. Nevertheless, the current range of peripheral physiology sensing devices faces several obstacles when it comes to effectively utilizing their data. For example, not all the available devices provide access to raw SC data, which are needed to design custom processing algorithms in research studies. Some commercial devices provide so-called stress indexes, which are derived from raw data by algorithms that are typically not disclosed by manufacturers. Being truly wearable means being able to wear something comfortably, and smartwatches provide a convenient and socially acceptable option. They have easily readable displays and can be customized through various apps and interconnected devices [64]. For scientists, these small wearable sensors are minimally intrusive and can be easily incorporated into a wide range of experimental designs. Additionally, they can be used over a long period of time by multiple individuals simultaneously and by vulnerable populations that are typically not well-represented in traditional neuroscience research. Furthermore, they are significantly more affordable compared with most neuroimaging equipment. Consumer-grade devices are remarkable engineering achievements that are priced affordably, making them accessible to a wide range of users. However, they have limited access to derivative data, which are calculated using undisclosed algorithms. This limitation restricts their usefulness for innovative scientific investigations.

When exploring the market of commercial portable and wearable devices capable of acquiring SC, which could be used in human monitoring applications, not so many options emerge; those available can be categorized as research devices and consumer-grade devices. One of the wearable devices most commonly used in research is the Empatica E4 (Empatica Inc., Cambridge, MA, USA), which is now going to be replaced by the newest Embrace one from the same manufacturer. Both are clinically validated devices (Class IIa Medical Device according to 93/42/EEC Directive), which can be used to measure the impact of different stimuli [68,69] or to classify elicited emotions [70]. Similar to the E4 device, Embrace hosts four sensors (a triaxial accelerometer, a skin thermometer, a triaxial gyroscope, and an SC sensor) and also provides several software tools to process the collected data on the Cloud platform where they are stored. Shimmer3 GSR+ (Shimmer Research Ltd., Dublin, Ireland) is another well-adopted device in research studies, thanks to the pre-amplified channel available for SC acquisition, which makes it possible to measure the electrical characteristics of the skin as well as an optical pulse/PPG signal on a different but synchronized channel for heart rate estimation. As research-grade devices, Empatica, Shimmer, and BioPac support extensive access to high-quality data.

However, these devices are priced at unaffordable levels. In fact, they offer access to raw data, which is different from consumer-grade devices, allowing in-depth analysis but with expensive licensing. Conversely, consumer-grade devices, conveniently worn on the wrist, only offer limited access to consumer-grade data derived through signal processing algorithms that are often not openly disclosed. Consequently, this can pose challenges when attempting to interpret the data within research contexts. The GoBe3 smart band (Healbe Corporation, Redwood City, CA, USA), for example, acquires a so-called emotional tension related to feelings and mood, somehow derived from the SC by analyzing the changes in cutaneous sweating. Additionally, three other embedded sensors are also provided, namely a bioimpedance sensor, an accelerometer, and a piezo sensor. Other consumer-grade devices that allow to acquire SC data are Fitbit Charge and Fitbit Sense (FitBit Inc., San Francisco, CA, USA), Amazfit Helio Ring (Amazfit, Hefei, China), Nuanic (Nuanic Oy, Tampere, Finland) and EmotiBit (Connected Future Labs, LLC, New York, NY, USA). Fitbit Charge and Fitbit Sense are devices equipped with an SC sensor that can measure the user stress level, and apps to provide insights on how to manage it. Amazfit Helio Ring and Nuanic are rings that can measure SC. Finally, EmotiBit is a device designed to provide an affordable solution that grants users access to raw data. Importantly, the data obtained from EmotiBit are fully owned by the user, ensuring privacy and control. Additionally, EmotiBit operates within an open-source ecosystem, allowing for flexibility and customization to explore and ask new types of questions about the physiological signals generated by bodies and minds. EmotiBit bridges the gap between research and consumer-grade devices by offering affordable access to raw data. This empowers users to delve deeper into their physiological signals and encourages the exploration of novel scientific inquiries [71]. Table 3 lists the commercially available wearable and portable devices for SC monitoring. It is interesting to notice they come in different options, from wrist-worn devices to portable acquisition systems to minimally invasive rings, usually intended for elite athletes, such as the Amazfit Helio Ring.

Klimek et al., in [72], analyzed several studies involving the acquisition of SC, identifying sixteen different wearable devices, among which the Empatica wristband was the most common (40.1% of the screened articles), followed by the Shimmer3 and Affectiva Q (Affectiva Inc., Boston, MA, USA) sensors. Overall, the vast majority of studies (almost 70%) used a wristband device, while a glove with integrated sensors was used in very few devices. Regarding the position of the electrodes, most of the studies referred to fingertips (Shimmer3 and BioPac systems, Neurobit Optima 4 (Neurobit Systems, Gdynia, Poland)), and a few experiments used palmar electrodes (Mindplace Thoughstream (Mindplace, Eastsound, WA, USA) or collected SC signals from the chest (using the RespiBAN Professional (PLUX wireless biosignals S.A., Lisbon, Portugal)), or the torso, by custom manufactured garment. Betancourt et al. [73] measured SC on the ankle using the Affectiva Q sensor.

### 2.4. PPG Sensors

Remote monitoring of subjects in AAL applications, where it is important to guarantee the personal safety of people who, perhaps due to age or cognitive disorders, could carry out risky activities, is certainly a research topic of crucial importance. Joint with activity monitoring and recognition, it is important to keep track of vital signs, such as blood oxygenation (SpO2), HR, or respiratory rate [74], while still allowing the person as much freedom of movement as possible [75]. Thus, it is important to collect and process a combination of data coming from heterogeneous physical domains, from which it is possible to extract much more significant and precise information than using just one type at a time. This can be done by exploiting photoplethysmography, an optical non-invasive measurement technique of peripheral blood flow commonly used to acquire parameters such as SpO2, HR, or heart rate variability (HRV).

A photoplethysmograph works by shining light, possibly at different wavelengths, through the skin of the person wearing the device and detecting the amount of light reflected or passed through, depending on the mechanical arrangement of the light source and detector. A green light source is commonly used for HR estimation from changes in blood volume during heartbeats, as this usually provides the highest signal-to-noise ratio. Both red and infrared light sources are instead used to measure blood oxygenation (oxygenated hemoglobin absorbs infrared light more and red light less, unlike de-oxygenated hemoglobin). Therefore, for greater flexibility, integrating all three types of light sources into the same sensor is the best choice for commercial and research devices that use the PPG signal and its second derivative, the acceleration plethysmography (APG) [76], for estimating HR and HR variations, as well as oxygen saturation and blood pressure [77,78,79,80].

When PPG signals are acquired in controlled clinical settings where sensors are often applied to the fingers, head, or earlobe, MAs are inherently weak. In this case, signal processing techniques based on time-frequency algorithms, such as empirical mode decomposition [81] and its modifications [82], or wavelets [83] and ad hoc filtering techniques, such as adaptive step-size least mean squares filter [84], can be successfully used.

In AAL applications where, instead, comfort and low invasiveness are important, PPG sensors are embedded in wearable devices that can be worn on the wrist, arm, or fingers, such as smartwatches, smart bracelets, or smart rings. Onboard these devices, miniaturized, low-power semiconductor technologies and multimodal signal acquisition and processing techniques have made it possible to obtain clean PPG signals by removing strong MAs derived from motion activities carried out by the people wearing them [85]. For example, a 2.76 mm^2^ chip that implements a light-to-digital converter for long-term continuous PPG monitoring was fabricated in TSMC 180 nm technology [86]. The widespread diffusion of these commercial wearable devices has also suggested the use of PPG signals alone [87,88] or, better, in combination with inertial signals [89] for the recognition of human activities, as it will be better explained in Section 3.5.

## 3. Integration and Processing of Wearable Sensors Data for AAL

As highlighted in previous sections, HAR is one of the most important functions of AAL systems and applications. HAR of the upper limb pursued by inertial data found widespread diffusion, but many other studies focused on the fusion of such kind of data with physiological signals able to provide further information regarding the upper limbs, hands, and finger movements since many activities performed in the daily living involve fine gestures, e.g., deployed for achieving grasping of different objects. Indeed, physiological biosignals, such as EMG and SC, are strongly related to HAR and within an AAL context, can provide additional, valuable information for the physiological state functional assessment of the user in terms of motor activity, mental stress, and, in general, human well-being. In particular, muscular activity recorded by surface EMG represents one of the major ways for HAR implementation. The following sections present different sensor integration modalities and related signal-processing approaches.

### 3.1. Integration of Inertial and EMG Sensors for HAR

The effectiveness of EMG signals, recorded with a sparse or high-density setup, for performing hand gesture identification using pattern recognition modalities is well-acknowledged [90]. Myoelectric activity represents one of the principal solutions for measuring and assessing physical activity in healthy and pathological individuals [91], but the widespread diffusion and availability of portable devices for EMG recording, such as armbands for forearm muscle recording [92], made this kind of biosignal a suitable solution for HAR in AAL applications. Indeed, EMG-based architectures have been extensively employed for developing human–machine interfaces for robotic exoskeletons and power prostheses [90]. However, they found extensive usage also for AAL-related applications [93], such as activity recognition, often in conjunction with inertial sensing data [94], affective computing, e.g., for micro-expression recognition and emotional response assessment [95], and eating behavior during the daily living [96,97,98]. Moreover, recent advancements in terms of probes design for biosignal recording [99] have allowed their embedding within garments or clothing, enlarging the field of applicability of the EMG signals toward home-based healthcare applications [42].

Integration of inertial sensors and EMG sensors was largely leveraged for hand gesture recognition, aimed at prosthetic and assistive device control. Shahzad et al. [100] used IMU information to make the myoelectric-based pattern recognition system aware of the position of the arm in order to enhance classification performances. Two magnetic, angular rate, and gravity (MARG) sensors were placed on the wrist and on the biceps brachii muscle and raw data were processed to obtain the orientation of the arm segments. Four EMG probes were placed instead on the flexor-extensor muscles on the forearm, and six hand motions were performed by volunteers during static and dynamic movements of the arm along defined trajectories. Thus, in this case, inertial information was leveraged to reconstruct the upper limb pose and not for feature extraction and pattern recognition. EMG and inertial data fusion, due to augmented information available, enhance the pattern recognition outcomes for gesture-related tasks, and thus, it was employed when a particularly challenging recognition objective must be achieved.

For instance, this configuration was explored for allowing the correct identification of sign language to enable communication and bridge the gap between deaf people and hearing people [51]. In the latter work, instead of using an instrumented glove, the forearm was chosen as the sensor location, and it was instrumented with four EMG probes and a single IMU sensor placed on the wrist, resembling a smartwatch location. From the latter, accelerometer and gyroscope data were considered. An adaptive auto-segmentation method was also proposed in order to potentially allow the system to work in real-time, based on the EMG signal energy computed on windows of 128 ms. Two different sets of features were extracted from myoelectric signals and inertial signals, outlining that the different nature of these kinds of data require dedicated feature selection to be computed. Overall, 9 features were computed on EMG and 13 from the tri-axial acceleration and angular velocity and from their magnitude, providing the dimensionality of the final feature space equal to 268. Classical machine learning models (DT, SVM, kNN, and NB) were used for classifying 80 sign language words repeated by four volunteers 25 times. Features were ranked separately for EMG and IMU channels in order to check which source of information was the most useful for the purposes of the study. Outcomes showed that acceleration was the most important modality in terms of feature ranking, followed by gyroscope data. EMG resulted in the least important, as confirmed also by the classification results, where the inclusion of EMG features only slightly improved the performance (about 4%), which with inertial data were only just above 90% for the 80 signs. However, EMG features improved the recognition of some specific signs that likely share some characteristics in terms of acceleration or angular rate, and thus, additional information was needed for correct identification. This indicates once more the value of inertial data for HAR, but also highlights that the selection of source information to be used within the classification pipeline is strongly dependent on the gestures or activities that are taken into account for a certain application.

### 3.2. Integrated HAR for Rehabilitation

Rehabilitation is an important field of research where multi-sensor fusion was explored. Song et al. [101] focused their efforts on the development of the wearable multimodal serious game for hand rehabilitation in stroke patients by proposing a multi-sensor fusion for hand movement classification involving IMU, force myography, and EMG. The system encompasses six EMG probes (Trigno wireless system (Delsys Inc., Natick, MA, USA)) placed on the forearm, eight barometric sensors, and one IMU mounted on the wrist. Hand activities considered for rehabilitative purposes involved twelve grasping tasks and forearm movements related to activities of daily living. Force myography and IMU were recorded at 36 Hz, whereas EMG data were collected at 1926 Hz. Features were computed on 200 ms windows with an increment of 50 ms, which is also suitable for real-time purposes. The mean absolute value was used as the only feature from IMU and force myography, together with eight additional features from EMG channels. LDA was employed as the preferred model for the classification of hand movements. In this case, IMU data alone showed the lowest performance, well below 60%, while the addition of EMG features raised the accuracy to about 75%, only slightly below the best configuration given by all three sources of information (80% accuracy). These results indicate that the selection of the appropriate source of information is crucial for HAR and is strongly dependent on the final objective of recognition. In this case, movements to be recognized were mainly hand gestures, which are more suitable for recognition by the myoelectric activity of the forearm. Indeed, EMG alone provided an accuracy comparable to that observed when both EMG and IMU data were used.

The same authors [102] investigated another setup, where two IMUs (MTw Awinda and Xsens (Xsens Technologies B.V., Enschede, The Netherlands)) were placed on the upper arm and the forearm in order to detect gross movements of the arm, whereas finer gestures of the wrist and hand were identified through six EMG probes (Trigno wireless EMG system) placed around the forearm. Also, in this case, eight barometric sensors (MPL115A2 and the Freescale semiconductor) were attached around the wrist. The system was composed of a serious interactive game for shoulder and elbow rehabilitation, and this is why gross movements needed to be identified. A total of six shoulder and elbow movements were considered, together with seven hand gestures. Features were only extracted from EMG and barometric sensors, whereas IMU data were used to measure the upper limb pose using quaternion estimation. This work highlights the wide potential of inertial data since they allow us to not only extract meaningful features from raw acceleration, gyroscope, and even magnetometer readings but also to obtain information related to the pose in the 3D space of the human segment where the sensor is mounted, thus significantly extending the type of information that can be retrieved from this kind of portable devices.

Eventually, it deserves to be mentioned that integration between inertial and EMG data was also leveraged by choosing the wrist as the location for IMU sensors, as in [103], where a hierarchical method for hand motion recognition was proposed, with a probe setup made by two EMG probes attached on the forearm, and a single IMU sensor mounted on the wrist, from which acceleration and gyroscope data were recorded for estimating device orientation. The proposed method showed high recognition accuracy, but it should be noted that only six common hand gestures were considered, mainly involving forearm and wrist motions, which can be relatively easily recognized when 3D pose data are available from an inertial device, outlining once more the potential value of a wrist-mounted IMU device for HAR related to the upper limb.

### 3.3. Integrated HAR for Industrial Applications

Due to their complexity, EMG and inertial data were also explored in the context of industry-related applications. For instance, in [104], an extensive dataset of working activities was collected using 17 IMU sensors (Xsens Technologies B.V., Enschede, The Netherlands) and 16 EMG channels prototyped in laboratory, located on the upper limb while performing seven activities that involve object manipulations, resembling industrial tasks. Human activities that involve full-body movements were also investigated for producing reliable recognition by integrating different sources of biosignals. In [105], a ResNetXt DL model, with three kernel sizes in each multi-kernel module, was proposed for the recognition of a series of activities related to walking, such as walking upstairs and downstairs, curving, spinning, running, and some others. A publicly available dataset was used in [106], where sensing technology encompasses IMU sensors embedding an accelerometer and gyroscope located on the thigh and shank, together with four EMG probes placed on thigh muscles and gastrocnemius. Although good results were achieved with the proposed learning scheme, the proposed setup can hardly be employed in a real usage scenario due to the high number of probes and multiple locations that could be uncomfortable to use on a daily basis.

Locomotion-related activities were also considered by Zhou et al. [107], whose objective was the recognition of four ambulation modalities, namely jumping, walking, ascending stairs, and descending stairs. For this purpose, eighteen volunteers were instrumented with three IMU sensors (sampling rate 100 Hz) and five EMG probes (sampling rate 1 kHz). IMUs were placed on the chest, the lateral side of the thigh, and the lateral side of the shank, whereas EMG electrodes were located on three thigh muscles and two calf muscles. A total of 15 features in multiple domains were extracted from EMG and acceleration and gyroscope signals, with 200 ms windows and an increment of 40 ms. Feature selection was performed by a Markov random field-based Fisher–Markov selector that minimizes the within-class distance of features and maximizes the between-class distance. Then, a series of ML models were compared (kNN, SVM, ensemble learning, and the back propagation neural network) with 10-fold cross-validation. Also, in this case, IMU-based features showed high discriminative capabilities, and the EMG feature only provided recognition accuracy less than 80% for the locomotion modalities, whereas IMU features showed about 90% accuracy for each classifier, confirming their value for this kind of pattern recognition problems, where similar human movement have to be correctly identified. However, sensor placement targeted the proposed pipeline for specifically recognizing walking-related movements, being hardly generalizable towards a different kind of movement, e.g., of the upper limb.

A focused analysis of inertial and myoelectric information was proposed in [108] in order to unveil if the compensatory balance responses needed for avoiding falls in free-living conditions are better detected with IMU, EMG, or a combination of them. Three Shimmer ExG wireless units with 3D IMU and two EMG probes were used in this study, and four additional EMG probes were used in this study, mounted on the shank, thigh, and sternum, whereas the myoelectric activity of two thighs and two shank muscles was recorded. Volunteers were asked to walk along a 10 m path, and they were randomly perturbed by lateral pushes to the right shoulder during the left and right swing phases of gait in order to deliver a side-step and a cross-over reaction, respectively. Then, features were extracted separately from IMU data and EMG data, and a set of hybrid features was also computed based on spatial and temporal distance between EMG and inertial signals. Three different kinds of classification tasks were tested involving the distinction between normal gait and perturbed gait and the type of perturbation, but also, in this case, outcomes showed that inertial information has the potential to be leveraged alone for recognizing such kinds of highly dynamic activities, outperforming EMG information.

### 3.4. HAR by Commercially Available Devices

In the past few years, devices such as armbands became commercially available for a variety of possible applications, from gaming to the interaction between humans and machines. In this field, the Myo armband (Thalmic Labs Inc., Kitchener, ON, Canada) was one of the first examples of this kind of technology, followed in more recent years by other devices such as the MindRove. In general, such wearable devices include several EMG probes and one IMU or MARG sensor embedded in a circular structure that should be worn by the subject on their forearm. Thus, a significant amount of information can be recorded since the disposition of the EMG probes allows us to record the forearm’s myoelectric activity, even if sensor location cannot be accurate, as this happens when gold-standard EMG probes are directly applied to the muscles of interest. Due to the novelty of the Myo armband, many studies tried to leverage this device, mainly for gesture recognition, due to the constraint of wearing the armband on the forearm. In [92,109], the Myo armband was leveraged for gesture recognition using only eight EMG probes, whereas in [110], the same device was used as a human–machine interface for the control and trajectory planning of a robotic arm; in this case, using EMG data and IMU as well but considering only a limited set of hand and wrist poses. A control system for the robotic arm was developed in the Simulink environment, and IMU data were used for obtaining the position and orientation of the robot. With an inverse kinematic analysis, the angular position of the robot joint was estimated to satisfy the desired end-effector trajectory. The effectiveness of the Myo armband for gesture recognition was also investigated in [111], where the addition of the IMU data was considered to address the problem of the multi-user usability of pattern recognition architectures since the latter represents a well-acknowledged problem in the field of myoelectric-based gesture and activity recognition. Further, the influence of different upper limb positions on the recognition of seven hand gestures was also explored. In this case, the improvement given by IMU data was strongly dependent on the specific ML model used for classification, highlighting, in this case, the non-negligible role of the pattern recognition architecture that has to be implemented.

The use of the Myo armband for recognizing human activities not limited to hand and wrist gestures was investigated in [112], where a feature selection method was employed for identifying the most meaningful features to recognize ten human activities, namely handshaking, hugging, bouncing, running, sitting, bending forward, standing, walking, sprawling, and applauding. However, in this case, only EMG data were processed, without relying on IMU data provided by the armband, and no classification was carried out. However, the investigation was limited to feature ranking. On the other hand, HAR by the Myo armband was exploited in [94], where a series of activities strongly related to the daily living scenario were considered. It is worth noting that the choice of the above-mentioned activities was based on their clinical relevance, on the basis of the functional arm activity behavioral observation system (FAABOS), a taxonomy to quantify the relevance of upper limb activities on various levels of significance [113]. Thus, a total of 17 activities were selected and grouped into four categories, i.e., non-functional activities, non-task-related activities, task-related activities, and high-exertion activities. EMG data were recorded at 200 Hz, whereas inertial data were recorded at 50 Hz. A set of simple time domain features were extracted from tri-axial acceleration, angular velocity, i.e., mean value, and magnitude area, whereas, for the eight-channel EMG, only the root-mean-square value was considered as a feature. Ten unimpaired volunteers were recruited for the study, who each performed the activity six times. As a pattern recognition algorithm, the kNN was used, where the number of neighbors was set as three. In this case, inertial data alone provided a promising 78.3% accuracy, outlining the value of this kind of data for HAR. As expected, the inclusion of myoelectric information boosted the performance to about 85%, but surprisingly, the best outcomes were observed when EMG was used in conjunction with gyroscope data, with 89.2% accuracy in recognizing all activities. Thus, the angular rate of change can be itself a valuable source of information for activities that involve upper limb motions, but this indicates once more that an a priori selection of data sources for HAR application is hardly feasible and can be accomplished only after preliminary evaluations, related to the specific kind of activities, are made. Finally, selecting the set of human activity based on recognized criteria, i.e., using the FAABOS taxonomy in this case, provides advantages in terms of study replicability and would allow the direct comparison of results obtained from different works, thus strengthening the field of HAR based on wearable devices. The FAABOS taxonomy is becoming an acknowledged basis for the choice of activities to be included in experimental setups when dealing with activity recognition in a scenario close to real contexts [114] because such kinds of movements are also meaningful from a clinical viewpoint.

Finally, it is interesting to note that integration between EMG and inertial data has also been employed for estimating the physiological state of individuals, such as fatigue. In [115], the Myo armband was employed to monitor fatigue in construction workers. In particular, the aim was the prediction of the aerobic fatigue threshold using EMG and inertial data of the forearm by estimating the average oxygen consumption that is related to the fatigue threshold by the maximum aerobic capacity. The fatigue threshold was estimated on windows of five minutes. In addition to the Myo armband, a metabolic analyzer and a heart rate monitor were also used, the former as ground-truth and the latter for comparative purposes. For classification, a bidirectional RNN-LSTM network was exploited. Fourteen scaffold-building activities were selected for eliciting fatigue in the ten recruited volunteers, with five different fatigue levels, and each activity was performed for no more than 10 min. The proposed methodology provided good results in terms of both the classification of the five levels of fatigue and the estimation of the threshold over time, suggesting the suitability of myoelectric and IMU data integration for monitoring fatigue levels over time.

The recognition of fatigue using inertial devices was also investigated in [116], where the focus was given to the gait task. Volunteers were asked to perform three different modalities: the first one was a common walking task alongside a straight path. The second one was a fatigued walking task where fatigue was elicited previously by a tiptoeing task and performed until the subject was no longer able to perform the task. Then, the same previously described walking task was performed. The cadence of the fatigued gait was recorded, and, on the second day, the subjects were asked to walk with a cadence equal to that recorded during the fatigue task, imposed by a metronome. Volunteers were instrumented with six IMU sensors (Trigno Avanti, Delsys Inc.) placed on the head, the second sacral vertebra, the left and right toes, and the heels. Additionally, six EMG probes were used for recording shank muscle activity and confirming the fatigued state of the subject by observing the median frequency over time and its descending rate. An LSTM network was used to classify the three gait modalities. The best performances were given by the combination of IMUs mounted on the toe and sacrum; IMU on the toes alone provided the best recognition of the three gait modalities (about 95%), whereas head-mounted devices showed the worst identification performances (around 60%). Although very promising results were shown in this study, the sensor setup required for the proposed application appears cumbersome for practical purposes, where the objective should be the reduction of the sensor’s number in order to avoid obtrusiveness and enhance user comfort. A summary of the works where inertial and myoelectric signals were fused for HAR is reported in Table 4.

### 3.5. SC and PPG Processing for AAL

As previously mentioned, the SC is measured to assess the sympathetic branch activity of the ANS that relates to attention and reaction to stress conditions. While the SCL is made up of the signal gradual tidal variation level and spontaneous signal changes, i.e., SCRs, also known as electrodermal responses (EDRs) or SC peaks, which are expressions of the subject’s reaction to specific stimuli.

A de facto standard method to process the SC signal is called trough-to-peak (TTP). The method searches for events in which the SC signal goes from a local minimum to a local maximum within a fixed response time window (typically 4 s). The SCR amplitude is defined as the difference between the SC values at the peak and at the preceding trough. A minimum amplitude criterion (e.g., 0.05 or 0.01 μS) is often applied to identify a peak. The TTP method is only used for analyzing acquired signals, and it does not rely on deconvolution, i.e., it does not require making an assumption about the shape of the SC peak signal generated by the underlying physiological reaction to a stimulus. A different approach decomposes SC data into SCL and SCR parts, the latter of which includes SC peaks that are of interest to analyze. This way, unsuperposed response components may be extracted to quantify SC peak characteristics in terms of amplitude and time distribution. SCR extraction may be performed according to so-called continuous decomposition analysis (CDA) or discrete decomposition analysis (DDA). The former aims at retrieving traces of the underlying ANS activity from the signal characteristics: SC data are deconvolved by a general response shape and then decomposed into continuous phasic and tonic components. The latter decomposes SC data into distinct phasic components and a tonic component by means of non-negative deconvolution, which is mainly aimed at studying the physiological models of the SC peaks. Both methods may also find application in the synthetic generation of SC signals by numerical simulation. Several free and open-source software (FOSS) are available for processing measured SC signals. Some of the most popular ones are OpenSignals (ver. 2.2.5), BioPatRec (2009 release), and OpenBCI GUI (ver. 6.0.0-beta.1). All of them can be used to record and analyze physiological signals such as SC, ECG, EMG, and more. They are also compatible with various sensors and devices and can be used for research and educational purposes.

While the responses of individuals in terms of SC and their physiological reactions may vary, wearable devices present an opportunity to customize treatment plans by providing real-time information on activities that either enhance or reduce arousal levels. This allows for targeted interventions aimed at specific attention-related and cognitive outcomes. Wearable technology is increasingly being tested in studies addressing the identification of personalized biomarkers in populations of subjects affected by anxiety, depression, or autism spectrum disorder (ASD) [64]. Remarkably, the acceptance and adherence to the use of wearables strongly depend on the body position they require to work, which is usually minimally invasive (e.g., wrist, hand, and fingertips) but not necessarily best accepted. In this regard, the proper positioning of SC electrodes to maximize the quality of the collected signals but also the user acceptance was investigated in [118] by comparing foot and fingers and in [119], where 16 different body locations were compared to measure the SC following emotional stimulation. Positioning electrodes on fingers was the choice resulting in the best signal quality, but finger sensors, which can be doable in laboratory conditions, may limit the freedom of subjects in daily life settings. It is interesting to notice that sensors in the form of jewellery-like rings, recently introduced to the market (e.g., the Nuanic smart ring), have not yet been extensively investigated for use in studies dealing with stress monitoring. Other body locations that exhibited good SC responsiveness in experiments are the forehead, shoulders, neck, chest, and even shoulders [119]. Wearable technologies can be developed for these bodily locations, making them more suitable for usage. The accuracy of devices to measure SC can vary depending on the device and the adopted method [120]. It is important to note that the accuracy of the device can be affected by various factors, such as the quality of the signal, the calibration of the device, and the placement of the electrodes [121].

Regarding the processing of PPG signals acquired by wearable devices outside of clinical contexts, as in most of AAL applications, ambient light and MAs sum up the semiconductor noise of the sensing element and greatly affect the collected signal quality in terms of the signal-to-noise ratio (SNR) and dynamic range (DR). Specific circuit blocks are consequently added to the sensor design to attenuate these interferences, further increasing power consumption. To extend the PPG sensor’s lifetime, ultra-low-power designs, circuit techniques, and sampling schemes have been proposed in the literature, as presented in [122]. The need for algorithms that meet real-time and low-power constraints for MA removal directly onboard the small and lightweight wearable devices commonly used for AAL applications has yielded excellent results in PPG acquisition while performing normal daily human activities [123]. A list of established real-time and low-power state-of-the-art methods used to remove MAs from PPG signals, which may or may not require the support of an additional IMU or a triaxial accelerometer embedded in the same device, is reported in Table 5.

Approaches based on the geometric separation of signal subspaces have also been presented in [136]. These have been designed to have a lower computational complexity than [131] while still offering comparable accuracy. Indeed, the dominant computational burden of these approaches lies in the SVD, which can be implemented leveraging any of many specialized algorithms [143,144], some of which are optimized for frequency tracking and so especially efficient in this context.

**Figure 1:**
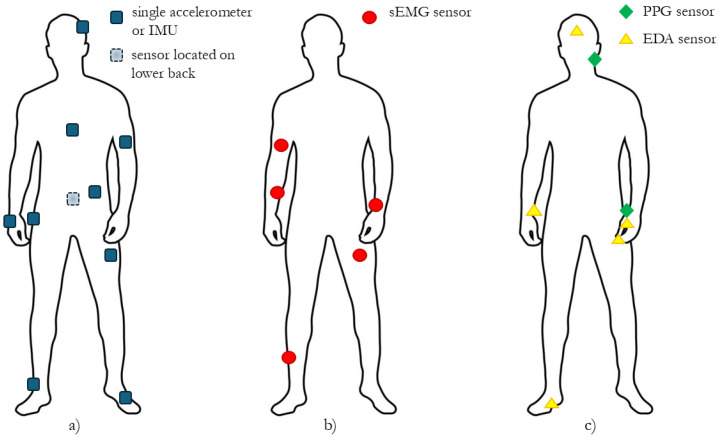
Typical body sites where sensors used in analyzed studies have been placed: (**a**) single accelerometer or IMU, (**b**) sEMG sensor, and (**c**) PPG and EDA sensors. Please note that the left or right side position of the marker representing the sensor is not relevant but only chosen for better readability of the figure.

Figure 1 provides a summary pictorial representation of the body sites where the above-mentioned and presented sensors are typically located according to the analyzed literature and commercial state of the art.

## 4. Discussion

The technological advancement of miniaturized sensors has allowed their embedding within mobile and wearable devices that can be continuously worn by subjects during daily living, potentially enabling a continuous monitoring of physiological state and motor habits. Physical activity represents one of the first aspects whose monitoring and assessment were performed by wearable devices, and applications included a wide spectrum of physical tasks, such as walking, standing, fall detection, rehabilitation, and physical activity recognition [91,145]. Although many other human behaviors and activities have been investigated in order to produce reliable monitoring through smart sensing, such as sleep, feeding behavior, and even human emotional condition by affective computing [93,146], the outbreak of the COVID-19 pandemic contributed to stressing two different aspects related to HAR. First, the importance of correctly identifying a series of activities related to human well-being and health, such as those activities related to personal hygiene [147]. Secondly, the value of technical solutions capable of remotely monitoring user activities, habits, and behavior within an in-home scenario was highlighted. Two common approaches are leveraged for HAR within a closed environment, i.e., wearable sensors and external or ambient devices, such as presence sensors and cameras. However, the latter approach exhibits two main potential drawbacks that can hamper a widespread diffusion for HAR purposes, namely the need for instrumenting homes with sensors and video devices and necessary data transmission capabilities, which in principle should be installed within each room where the user is supposed to perform the activities of interest. This would lead to undesired additional costs for a private user that cannot always be faced. Secondly, video cameras record images not only of the internal space of the room but also of the subject and bystanders, implying several issues regarding privacy aspects, even about storing this kind of sensitive information [148]. On the other hand, wearable devices provide information related to the activities performed, which prevent any direct identification of a specific user, and can be processed with small computational delay, thus potentially allowing also an online identification of specific activities performed by the subject.

When testing a wearable device-based scheme for HAR, by leveraging portable devices that can include EMG and inertial sensors, the aim should be developing a solution that would be able to correctly identify activities of daily living that mainly involve upper limb and hand, with particular focus on some human activities that have a specific role for subject well-being and health. While conceiving such kinds of systems, many different experimental and technical points deserve attention since they can lead to different final framework configurations, each of them tailored for specific applications in the field of HAR.

The first point regards the human activities to be recognized by the system and those activities that should be included in the whole set. As outlined before, a wide spectrum of possibilities is available in this case, ranging from activities involving full body movements, such as walking, running, standing, lying, and many others [145], to those that mainly rely on upper limb motion, including interaction with objects, and several kinds of manipulations [149]. For the purposes of AAL, many activities to be considered involve upper limbs and can be performed commonly during daily living. The rationale for this choice is based on their importance for many aspects related to health and well-being: for instance, the correct identification of the drinking gesture can be useful for monitoring fluid intake and hydration levels [150], and the recognition of bringing the hand to the mouth with a certain grasping shape can be linked to pill intake, useful for medical adherence assessment [151], and also eating. Thus, specific attention shall be given to such kinds of gestures and activities, and within the selected set, other daily living activities that can have similar execution mechanics should be included in order to test the robustness of the recognition methodologies to potentially confounding activities. However, it should be noted that the inclusion of additional activities that involve the whole body movement can be considered as well in order to enlarge the spectrum of detectable activities through the specific sensor configuration chosen. The latter aspect is another point of primary importance since it strongly impacts the overall performance of HAR systems. Many possible locations for sensor placement on the human body are possible, but the focus on the upper limb naturally drives the choice towards three main possible locations, i.e., the forearm, the wrist, and the hand. Although the forearm represents perhaps the most preferred choice for gesture recognition of the upper limb when also EMG information is leveraged [90], from a practical viewpoint, this configuration is not optimal since wearing sensors or even a smart device like an armband continuously during the day could be hardly acceptable by a user, being an unnatural condition. The latter aspect is also valid for the hand, where the presence of sensors would likely modify the physiological way of making gestures. Therefore, the wrist appears to be a preferred location for sensor placement, also considering that some previous studies have already investigated HAR with wrist-mounted devices [151,152] and that smart devices embedding miniaturized inertial sensors, such as smartwatches, have widespread diffusion and are now well accepted by users who can wear this kind of devices for the entire day and sometimes even during the sleeping periods. However, attention will also be devoted to the forearm location in order to establish a benchmark for comparing the performances of HAR attained by wrist-mounted sensors. The focus on the wrist for sensor placement also leads to the choice of leveraging mainly inertial information for HAR. This is justified in part by the fact that myoelectric probes are not available on commercial devices, even if some attempts have been made to develop wrist-mounted EMG sensors [153]. However, more importantly, myoelectric information that can be retrieved from the wrist is of poorer quality with respect to that from the forearm, where muscles responsible for wrist and finger movements can be easily recorded with surface EMG probes.

It is worth noting that even if a single inertial unit is used, different information can be extracted and then processed from this kind of device since each embedded sensor provides tri-axial measurements of acceleration and angular velocity, in the case of an IMU, and also of the magnetic field, in the case of a MARG sensor. This means that different characteristics related to movements and activities are potentially available from nine channels using only a single sensor, thus providing significant information for feature extraction and training of learning models for automatic HAR. In addition, 3D orientation of an inertial sensor can be estimated, also in real-time, by different algorithms [154] in the form of Euler angles or quaternions, thus representing further information that can be used for HAR purposes. Additional points that could be investigated within the proposed solution are related to the artificial intelligence pipeline that has to be employed for classification. In this field, two main aspects should be taken into account, namely the feature extraction and the classification architectures. In the first case, points that can be investigated are related to the optimal windowing for feature extraction, which is strongly related to the temporal duration of the various activities and different schemes for feature extraction since recently, various techniques have been proposed that also account for temporal and spatial dependencies between signals [90], rather than a more straightforward feature extraction in time and frequency domain with fixed window length. Regarding classification methodologies, relatively simple machine learning architectures have found widespread usage in the field of HAR, having low computational expense that makes this kind of model particularly attractive to applications that must be run online. However, comparisons between different ML models have shown that their performances can vary depending on the considered application [152,155]; thus, finding the best classification model for the selected sensors setup and activities to be recognized represents one of the potential points to be investigated when dealing with the pattern recognition aspects of a HAR framework. On the other hand, unsupervised approaches, such as deep learning models, would be also worth investigating in this context, since they would allow us to avoid handcrafted feature extraction by relying directly on sensors’ raw data. Further, this kind of methodology showed a generally good performance for HAR based on inertial data only, often outperforming machine learning architectures [151,156]. However, deep learning for pattern recognition has some drawbacks related to the amount of data required for model training and validation, higher computational costs with respect to machine learning models, and also the fact that deep learning models are, in general, black box-like models for feature extraction, which can limit the interpretability of the learned features for the final pattern recognition, and for the extraction of meaningful and relevant information for the purpose of AAL.

## 5. Conclusions

This paper focused on the importance of wearable devices in the design of AAL systems and applications, with a specific discussion of the advantages they provide in capturing information regarding human activities performed in uncontrolled scenarios and physiological parameters pertaining to motor capabilities, health status, and emotional well-being. In fact, the advances in electronic components’ miniaturization in addition to increased signal processing efficiency have led to the widespread adoption of commercial wearable devices that well beyond the originally targeted applications of fitness or leisure activities.

Despite the potential for groundbreaking discoveries, wearable technology has its limitations. Many wearable devices currently available on the market utilize proprietary algorithms and do not provide access to raw data. Consequently, claims cannot be fully verified, research inquiries are restricted to the available aggregated data, and it becomes challenging to distinguish actual data from artifacts. Additionally, the performed analysis of the literature shows that relevant information about sensor selectivity and sensitivity may affect the quality of the acquired signals, which is, in turn, reflected in more accurate classification of activities performed or better detection of parameters of interest related to the subject being monitored, is typically missing in research studies, but also unavailable for many commercial devices as well. Implementing HAR techniques using wearable devices into AAL solutions should thus turn our attention to a set of specific activities commonly performed during daily living within a home scenario, focusing on a specific subset whose recognition would be valuable for the monitoring of various aspects related to the health and well-being of a user. The measurement setup should be minimized as much as possible in order to improve the comfort of the user, the portability of the proposed technology for continuous monitoring during the day, low power consumption, and the extended lifetime of the device.

## Figures and Tables

**Table 1:** Experimental setup information of works using wearable accelerometers for human activity recognition (SR stands for sampling rate).

Reference	Number, Type, How It Is Used	Sensor Characteristics	Sensor location	Activities Considered for Recognition
Rodriguez-Martin et al. [16]	single, triaxial, used alone	model LIS3LV02DQ, ±6 g full scale, SR: 200 Hz	wearable, on waist	walking, stand, sit, lying, sit to stand, stand to sit, bending up/down, lying from sit and sit from lying transitions
Vakacherla et al. [17]	single, triaxial, used alone	−2000 to 2000 cm s^−2^ full scale, SR: 200 Hz	wearable, on user chest	standing, walking on level ground, walking on an incline, running, squatting
Tian et al. [18]	single, triaxial, used alone	±6 g full scale, SR: 150 Hz	wearable, on waist	sitting, jumping, walking, standing, lying, running, ascending stairs, descending stairs
Meng et al. [19]	single, triaxial, used alone or joint with gyroscope and sEMG	SR: 148 Hz for accelerometer and gyroscope, SR: 1260 Hz for sEMG sensor	wearable, on waist	walking, tooth brushing, face washing, drinking
Mannini et al. [20]	two, triaxial, used independently	±4 g full scale, SR: 90 Hz	wearable, on dorsal aspect of dominant wrist and on outside of the ankle	many (23 to 26) laboratory-based physical activities and common daily activities, grouped into four classes: sedentary, cycling, ambulation, other activities
Mannini et al. [21]	five, biaxial, on-body network	SR: 76.25 Hz	wearable, on: hip, wrist, arm, ankle, thigh	sitting, lying, standing, walking, stair climbing, running, cycling

**Table 2:** Summary of studies describing miniaturized sensors, and sensors able to perform multimodal signal acquisition for different monitoring purposes.

Reference	Acquisition Capability	Acquired Signals	Notes
Doheny et al. [40]	multimodal, compressed sensing and wireless powering capabilities	EMG, ECG	battery-free, standard 0.18-μm CMOS technology occupying a silicon area of 4.25 mm^2^
Dow et al. [36]	multimodal	EMG, ECG, vibration, temperature	analog front-end in 180 nm CMOS technology, applied in elderly care and precision sports study
Orguc et al. [34]	modular, low-voltage, ultra-low-power (3.8 nW)	EMG	65 nm CMOS technology, on 0.22 mm^2^ silicon area
Jani et al. [46]	multimodal, low-power	EMG, ECG	8.85 cm^2^ sensor readout PCB area, sensor weight ≤ 10 g
Biagetti et al. [47]	multimodal, three channels, low-power	EMG, ECG, IMU	13.77 cm^2^ sensor readout PCB area, sensor weight: 40 g
Said et al. [48]	multimodal, real-time data acquisition	EMG, ECG, triaxial acceleration, electrodermal activity (EDA), temperature	used to implement real-time monitoring systems
Pinto et al. [49]	multimodal	sEMG, ECG, triaxial acceleration	used in aquatic environments
Zhang et al. [50]	multimodal	five channel EMG, triaxial acceleration	used in hand gesture recognition
Wu et al. [51]	multimodal, real-time data transmission or local storage	four channel EMG, nine-axis motion sensor	100 Hz sampling rate for inertial signals, IEEE 802.15.4 wireless module
Tanweer et al. [52]	multimodal	EMG, ECG, IMU	to be applied on user chest, inertial sensor signals used for motion artifact removal
Das et al. [53]	multimodal	EMG, ECG, electrooculography (EOG), EEG	custom-designed ultra-low-noise instrumental amplifier used as an analog front-end with both programmable gain and bandwidth
Ding et al. [54]	hybrid (electrophysiological, acoustic, optical)	sEMG, mechanomyography (MMG), near-infrared spectroscopy (NIRS)	used to study interaction among grip strength, blood oxygen metabolism, and acquired signals
Ke et al. [55]	modular and multimodal, onboard signal conditioning and amplification	EMG, forcemyography (FMG)	floating electrodes used, A/D signal conversion at the base station

**Table 3:** Comparison of commercial wearable and portable devices for EDA monitoring.

Device	Measured Quantity	Sampling Frequency	Availability of Raw Signal Samples	Type of Device
Amazfit Helio Ring	skin conductance	not provided	no	wearable
BioPac MP36R (BIOPAC Systems, Inc., Goleta, CA, USA)	skin conductance	500 Hz	yes	portable
EmotiBit	skin conductance	not provided	yes	wearable
Empatica Embrace	skin conductance	4 Hz	yes	wearable
Fitbit	skin conductance	not provided	no	wearable
GoBe3	skin resistance	10 Hz	no	wearable
Nuanic	skin conductance	not provided	no	wearable
ProComp Infiniti	skin conductance	256 Hz	yes	portable
Shimmer3 GSR+	skin resistance	32 Hz	yes	wearable

**Table 4:** Summary of experimental setup information from works in which the fusion of inertial and myoelectric data was applied for human activity recognition.

Reference	Type and Number of Sensors	Probes Location	Raw Inertial Signals	Activities	Sensors Characteristics
Totty and Wade [94]	Myo Armband (8 EMG probes and 1 IMU)	Forearm	Triaxial ACC and GYRO. Feature extraction	4 categories of FAABOS activities	SR: 50 Hz (IMU), 200 Hz (EMG)
Shazad et al. [100]	4 EMG probes and 2 MARG	Forearm and upper arm	Triaxial ACC, GYRO, and MAG. Pose estimation	Six hand gestures for six forearm positions	SR: 1 kHz (EMG)
Wu et al. [51]	1 MARG and 4 EMG probes	Forearm (EMG) and wrist (MARG)	Triaxial ACC and GYRO. Feature extraction	80 ASL	SR: 100 Hz (MARG), 1 kHz (EMG), RES: 0.4 μV (EMG)
Bassani et al. [104]	17 MARG and 4 EMG probes	Full body (MARG), upper arm and forearm (EMG)	Triaxial ACC, GYO, and MAG	8 working activities	Full scale ±160 ms^−2^ (ACC), ±2000 °s^−1^ (GYRO), SR: 240 Hz (MARG), 500 Hz (EMG)
Liu et al. [106]	4 EMG probes and 2 IMU	Thigh, shank	Triaxial ACC, GYRO	22 walking-related activities	SR: 1 kHz (EMG), 100 Hz (IMU), RES: 16 bit
Zhou et al. [107]	5 EMG probes and 3 IMU	Chest (IMU), thigh (EMG and IMU), shank (EMG and IMU)	Triaxial ACC and GYRO. Feature extraction	4 walking-related activities	SR: 1 kHz (EMG), 100 Hz (IMU)
Nouredanesh and Tung [108]	3 MARG and 4 EMG probes	Chest (IMU), thigh and shank (EMG and IMU)	Triaxial ACC and GYRO. Feature extraction	Detection of compensatory balance responses	SR: 512 Hz (EMG, IMU)
Song et al. [101]	6 EMG probes, 1 MARG, 8 barometric pressure sensors	Forearm (EMG), wrist (MARG, barometric sensors)	Triaxial ACC, GYRO, and MAG. Pose estimation	11 hand gestures for rehabilitation with serious game	SR: 1926 Hz (EMG), 36 Hz (MARG, barometer)
Song et al. [102]	2 MARG, 6 EMG probes, 8 barometric pressure sensors	Forearm (EMG, MARG), upper arm (MARG), wrist (barometric sensors)	ACC, quaternions. Pose estimation and feature extraction	6 gross arm movement and 7 hand gestures	SR: 2 kHz (EMG), 90 Hz (barometer), 40 Hz (MARG)
Chang et al. [103]	2 EMG probes, 1 IMU	Forearm (EMG), wrist (IMU)	Triaxial ACC and GYRO. Segmentation and feature extraction	6 hand gestures	SR: 1.5 kHz (EMG), 50 Hz (IMU)
Biagetti et al. [117]	3 EMG and acceleration probes	Upper arm	Triaxial ACC. Feature extraction	4 strength exercises	SR: 2 kHz (EMG, ACC), full scale ±12 g (ACC)
Schabron et al. [110]	Myo Armband (8 EMG probes and 1 IMU), 1 IMU	Forearm (EMG), robot end-effector (IMU)	Triaxial ACC and GYRO. Pose estimation	5 hand gestures for robotic control	SR: 50 Hz (IMU), 200 Hz (EMG)
Colli Alfaro and Trejos [111]	Myo Armband (8 EMG probes and 1 IMU)	Forearm	Triaxial ACC and GYRO. Feature extraction	7 hand gestures in 4 arm positions	SR: 50 Hz (IMU), 200 Hz (EMG)
Bangaru et al. [115]	Myo Armband (8 EMG probes and 1 IMU)	Forearm	Triaxial ACC and GYRO. Feature extraction	Estimation of 5 levels of fatigue	SR: 50 Hz (IMU), 200 Hz (EMG)
Lee et al. [116]	6 IMU, 6 EMG probes	Shank (EMG), Head, lower back, feet (IMU)	Triaxial ACC and GYRO. Feature extraction	Fatigue identification	Not reported

ACC: accelerometer; ASL: American sign language; FAABOS: functional arm activity behavioral observation system [113]; GYRO: gyroscope; MAG: magnetometer; SR: sampling rate.

**Table 5:** Summary of real-time and low-power processing approaches to remove artifacts from PPG signals with and without the use of an additional accelerometer.

Reference	Artifact Removal Technique or Method	Target Artifact	Additional Accelerometer Needed?
Kim et al. [124]	independent component analysis combined with block interleaving and low-pass filtering	MA	Yes
Foo [125], Gibbs et al. [126]	adaptive filtering	MA	Yes
Tanweer et al. [127]	reference generation using singular value decomposition and multistage application of filtered X-LMS algorithm	MA during jogging	Yes
Chowdhury et al. [128]	multiple reference adaptive noise cancellation algorithm	MA, by four versions of cleaned PPG signal	Yes
Galli et al. [129], Lee et al. [130]	Kalman filtering	MA, improves HR tracking from PPG signal	Yes
Zhang et al. [131]	general framework tailored to hardware design	extremely strong MAs, improves HR estimation	Yes
Zhang et al. [132]	JOint Sparse Spectrum reconstruction (JOSS)	extremely strong MAs, improves HR estimation with no extra signal processing needed	Yes
Islam et al. [133]	cascade of adaptive, recursive-least-squares filters, aided by singular spectrum analysis	MA, improves HR tracking	Yes
Zhang et al. [134]	spectrum subtraction and empirical mode decomposition	MA, improves HR changes tracking	Yes
Islam et al. [135]	spectrum subtraction and empirical mode decomposition combined with a higher-accuracy wavelet-Fourier frequency estimator	MA, improves HR changes tracking	Yes
Biagetti et al. [136]	geometric separation of signal subspaces complemented with an automatic activity intensity classifier	extremely strong MAs, improves HR estimation	Yes
Zhao et al. [137]	short-time Fourier transform and statistical-based tracking algorithm	MA	No
Brophy et al. [138]	convolutional neural network without inertial data	MA in HAR and HR estimation	No
Dubey et al. [139]	specialized approach	MA in HR monitor during running	No
Wójcikowski et al. [140]	time-domain approach	MA in HR monitoring	No
Temko et al. [141]	Viterbi-based tracker in offline processing	MA in HR monitoring	No
Cajas et al. [142]	model of PPG noise components from database of corrupted PPG signals	MA in HR monitoring	No

## Data Availability

No new data were created or analyzed in this study. Data sharing is not applicable to this article.
